# Annotations

**Published:** 1902-08-09

**Authors:** 


### ANNOTATIONS.

### A Record Hospital Sunday in London.

The results of Hospital Sunday in London in the Coronation year are as remarkable as they are satisfactory. Never before in the history of the Sunday Fund had the influences which make against large contributions to the hospitals in the churches been so serious. People were never so insistently dunned for contributions to charities as they had been this year; moreover, the meeting in aid of the Coronation Gift was fixed for the Monday in Hospital Sunday week. Thus the fact that, notwithstanding these competing influences, a record Hospital Sunday collection was made speaks volumes for the loyalty and interest of all the churches, as well as for the efficiency of the new secretary, Sir Edmund Hay Currie. A word of congratulation is due to the founders of Hospital Sunday in London. Foremost amongst them were the late Dr. Wakley of the *Lancet*, Sir Sydney Waterlow, and the late Canon Miller, with one or two veterans who are still members of the Council. The late Dr. Wakley's robust faith in the possibility of uniting Londoners in the cause of suffering on one Sunday in the year, and his love for the hospitals, have this year borne fruit in the form of a collection amounting to no less than £62,000. Such a result must have given infinite pleasure to his brother, Mr. Thomas Wakley, who was present at the meeting, and who, though advanced in years, is a splendid specimen of robust humanity, and a remarkable representative of the Wakley family. All honour to them, and to all who have helped this good cause. Never before have the metropolitan press rendered such united and effective service to the hospitals, and the result will, we hope, encourage our contemporaries to continue their efforts year by year. But the chief honour is due to the clergy and ministers of religion and their congregations, seeing that the majority of the collections in more than eighteen hundred places of worship show an increase and in many cases mark a record. This remarkable result testifies to the confidence of the public in the management of the Sunday Fund. It is difficult to adequately express our admiration for the splendid liberality and noble example set to Londoners by Mr. George Herring, whose contributions in four years to the hospitals through the Hospital Sunday Fund have exceeded £40,000. These magnificent gifts have done much more good than mere money can yield, by being made in such a form as to call forth larger gifts from others. We rejoice, as all London must rejoice, that by the united efforts of preachers and people, press, philanthropists and prominent financiers, Hospital Sunday 1902 has made a "record," an event which will go down in history as amongst the most remarkable incidents in the year of King Edward's Coronation.

### The Efficacy of Revaccination.

The account given by Dr. R. S. Thomson and Dr. R. Fullarton of the cases of small-pox treated in Belvidere Hospital, Glasgow, during the outbreak of 1900–1901 contains interesting evidence of the prophylactic power of revaccination, evidence moreover of such a kind as to appeal to those who fail or refuse to comprehend statistics expressed in decimals and percentages. Here everything is indeed "marked in plain figures." Not one of the six revaccinated van men who were regularly engaged in bringing supplies to the hospital took small-pox; "but on one occasion a substitute who had not been revaccinated was sent in place of one of these. This man paid only one visit to the hospital, and was admitted a fortnight later with small-pox." Again, during the progress of the epidemic it was found necessary to erect temporary pavilions, in doing which 230 workmen were employed. Of these 217 were successfully revaccinated, all of whom escaped small-pox. Of the 13 however, who, for one cause or another, were not revaccinated, five were attacked with small-pox and one died. Here we have evidence that the escape of the whole of the revaccinated staff of nurses was not due, as the anti-vaccinists sometimes assert to be the case, to the sanitary conditions of hospital life lessening the chances of infection. Evidently the whole place swarmed with infection, which, however, virulent as it proved itself in the case of adults who had not undergone revaccination, was harmless in the case of the revaccinated staff.

### Porous Walls.

The old story about the advantages accruing from the walls of dwelling houses being porous has been revived again and it has been stated with some semblance of authority by certain writers that the employment of reasonably porous building material should be advocated in all new constructions, the suggestion being that the ventilation which takes place through such walls is not only appreciable, which of course is well known, but is advantageous. The advocates of ventilation by transpiration seem however to have overlooked a detail which is almost as well known as the permeability of brick and plastered walls namely, that such walls act as filters to the air and like all other filters tend to "clog." In ventilation by "transpiration" an exchange takes place, air passing outwards as well as inwards, and if this outgoing air deposits its impurities in the interstices of the wall through which it filters, which it certainly does, we arrive at this that a porous wall soon becomes a foul wall. There can indeed be little doubt that the unhealthiness of some old houses is partly due to the accumulation of the dust of ages deposited in every chink and cranny which is inaccessible to the duster. Flooring loosely laid allows dust, spiders and beetles to get through, so that the space between floors and ceilings becomes a foul receptacle for dirt of many kinds including the corpses of endless generations of insect life. Behind the skirting boards the same process takes place, dead mice forming a somewhat prominent item in the collection, and in porous walls again the same process of accumulation is in constant operation, only the things accumulated are smaller. If we are to get rid of "stuffiness" in our houses we must get rid of all these receptacles for dirt—of porous walls among the rest. The inlets by which fresh air is admitted should be as direct and open as possible, so that the air shall come in clean and pure. Far better to breathe a few smuts—which after all have been purified by fire—than to breathe air which has been filtered through bricks charged with impurities gathered up during years of use.

